# cDNA-detector: detection and removal of cDNA contamination in DNA sequencing libraries

**DOI:** 10.1186/s12859-021-04529-2

**Published:** 2021-12-24

**Authors:** Meifang Qi, Utthara Nayar, Leif S. Ludwig, Nikhil Wagle, Esther Rheinbay

**Affiliations:** 1 Center for Cancer Research, Massachusetts General Hospital, Charlestown, MA 02129 USA; 2 Harvard Medical School, Boston, MA 02115 USA; 3 Broad Institute of MIT and Harvard, Cambridge, MA 02142 USA; 4 Department of Medical Oncology, Dana-Farber Cancer Institute, Boston, MA 02215 USA; 5 Department of Pathology, Massachusetts General Hospital, Boston, MA 02114 USA; 6 Present Address: Department of Biochemistry and Molecular Biology, Bloomberg School of Public Health, The Johns Hopkins University, Baltimore, MD USA; 7 Present Address: Department of Oncology, Sidney Kimmel Comprehensive Cancer Center, Johns Hopkins University School of Medicine, Baltimore, MD USA; 8 Present Address: Berlin Institute of Health at Charité – Universitätsmedizin Berlin, 10117 Berlin, Germany; 9 Present Address: Max‐Delbrück‐Center for Molecular Medicine in the Helmholtz Association (MDC), Berlin Institute for Medical Systems Biology (BIMSB), 10115 Berlin, Germany

**Keywords:** Contamination, Genomics, Software, Quality control, cDNA

## Abstract

**Background:**

Exogenous cDNA introduced into an experimental system, either intentionally or accidentally, can appear as added read coverage over that gene in next-generation sequencing libraries derived from this system. If not properly recognized and managed, this cross-contamination with exogenous signal can lead to incorrect interpretation of research results. Yet, this problem is not routinely addressed in current sequence processing pipelines.

**Results:**

We present cDNA-detector, a computational tool to identify and remove exogenous cDNA contamination in DNA sequencing experiments. We demonstrate that cDNA-detector can identify cDNAs quickly and accurately from alignment files. A source inference step attempts to separate endogenous cDNAs (retrocopied genes) from potential cloned, exogenous cDNAs. cDNA-detector provides a mechanism to decontaminate the alignment from detected cDNAs. Simulation studies show that cDNA-detector is highly sensitive and specific, outperforming existing tools. We apply cDNA-detector to several highly-cited public databases (TCGA, ENCODE, NCBI SRA) and show that contaminant genes appear in sequencing experiments where they lead to incorrect coverage peak calls.

**Conclusions:**

cDNA-detector is a user-friendly and accurate tool to detect and remove cDNA detection in NGS libraries. This two-step design reduces the risk of true variant removal since it allows for manual review of candidates. We find that contamination with intentionally and accidentally introduced cDNAs is an underappreciated problem even in widely-used consortium datasets, where it can lead to spurious results. Our findings highlight the importance of sensitive detection and removal of contaminant cDNA from NGS libraries before downstream analysis.

**Supplementary Information:**

The online version contains supplementary material available at 10.1186/s12859-021-04529-2.

## Background

Massively parallel DNA sequencing experiments including ChIP-seq, ATAC-seq, whole exome and whole genome sequencing are widely used research methods. These methods have been instrumental for identifying binding sites of DNA-associated proteins, histone modification states, chromatin accessibility, and germline and somatic genomic variants. In common functional studies, a wild-type gene or variant of interest is introduced into the experimental system with DNA vectors, which can then appear in the sequencing library. Sequence reads stemming from the exogenous gene of interest are then mapped back to the endogenous locus during the alignment step. In addition, trace amounts of vector DNA from other experiments performed in the same laboratory or on shared equipment can contaminate the DNA library, leading to the presence of foreign genes in the derived alignment. Signal from these contaminant genes can then affect downstream results and interpretation, for example through spurious increased copy number [1, 2], false germline or somatic variant calls [3, 4], or incorrect coverage peak calls [3–5].

Detection of such exogenous gene signal in an alignment can be challenging, in particular when the vector-derived signal overlaps true signal derived from exome or chromatin profiling. Few methods have been developed to detect vector contamination in NGS libraries. Some identify sequence reads, such as SeqClean and VecScreen [6, 7], from known cloning vectors, but do not search for the cDNA insert. Vecuum [4] was developed to also identify the contaminating gene; however, this tool relies on known vector backbone sequences.

**Fig. 1:**
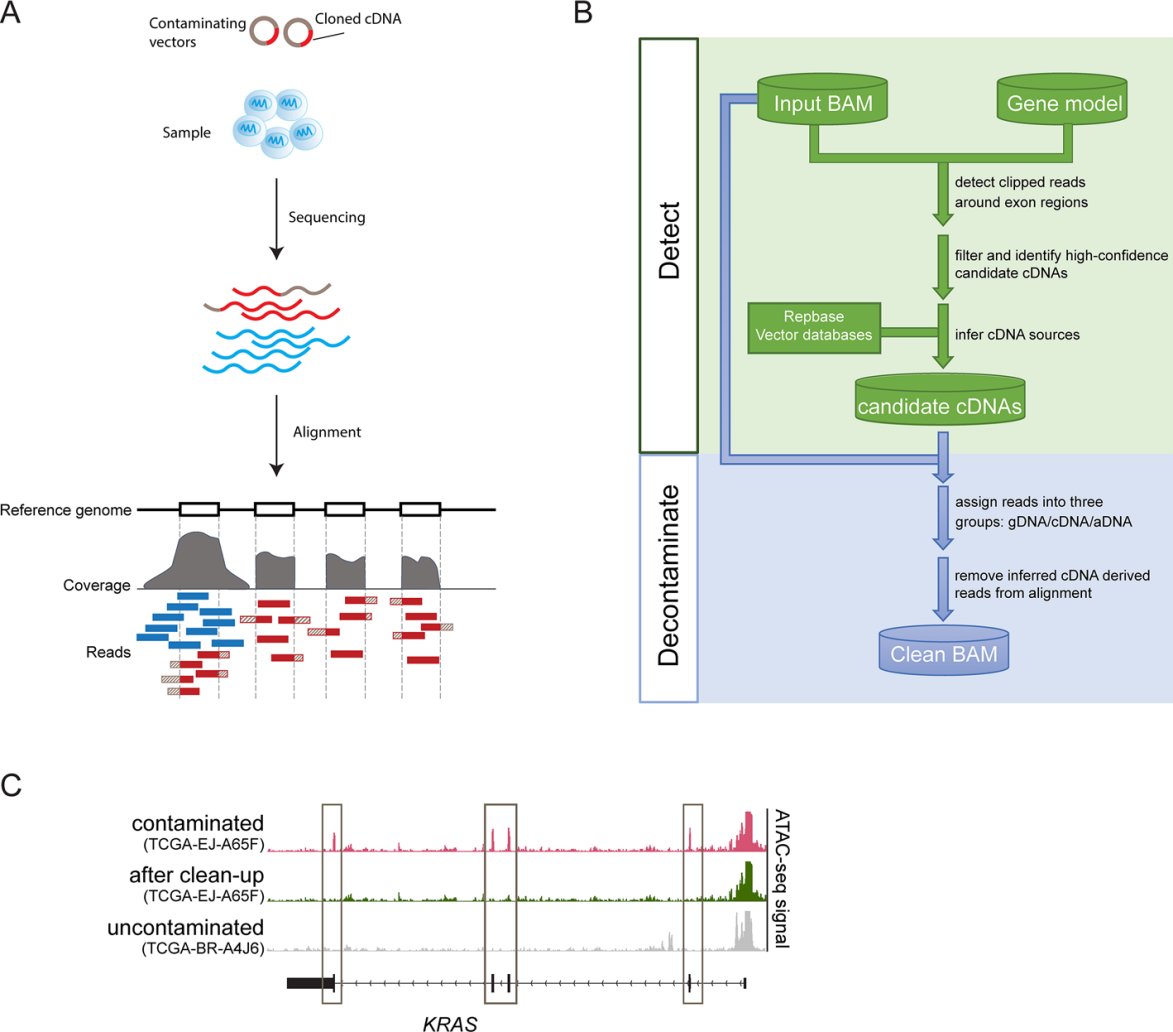
Workflow of cDNA-detector. **A** Schematic illustrating the source of intentional or contaminant cDNA reads in sequencing experiments. Vectors (grey) with cDNA (red) are amplified in the library preparation process and sequenced together with the experiment. Upon alignment of reads, cDNA-derived reads (red boxes) map to the respective gene locus in the genome, along with true signal reads (blue). Textured red read segments indicate sequence not mapping to the genome and “clipped” by the alignment algorithm. **B** Overview of the two main components of the cDNA-detector algorithm, “detect” and “decontaminate”. **C** ATAC-seq experiment from TCGA before (pink) and after (green) removal of vector-introduced *KRAS*. For comparison, an uncontaminated sample (grey) is shown. Boxes indicate contaminant signal over exons

Because cloned cDNAs do not contain introns or their physiologic UTR regions, this property can be exploited to identify potential contaminants. Reads from these cDNAs only partially align to the genome at exon boundaries, with unmapped sequence matching either a vector (at the 5’ and 3’ ends) or a neighboring exon. In a genomic alignment, these reads appear as “clipped”, and thus can be distinguished from true signal reads that fully map to the genome across exon boundaries (Fig. 1A).

## Implementation

### Detection of cDNA in NGS libraries

Prompted by the presence of intentional and accidental cDNA cross-contamination in our own data, we developed cDNA-detector, a computational tool that detects and optionally removes cDNA contamination in DNA sequencing data (Fig. 1B). cDNA-detector takes a standard BAM-formatted alignment and a gene model file as input. For each exon from the model file, the number of clipped and properly mapped reads at the two boundaries (the start and end coordinate) are counted. Using a binomial model, we test whether the fraction of clipped reads at any given boundary coordinate exceeds the expectation based on the background of total clipped reads (Methods). The two *P*-values for each exon and all *P*-values for each transcript are combined. Significant transcripts (*P* < 0.05) or transcripts with more than 30% of significant exons are considered candidate contaminant transcripts for further analysis (Additional file 1: Figure S1).

Next, cDNA-detector performs an in-depth search for additional evidence at exon boundaries for all exons in candidate contaminant transcripts. First, it seeks clipped reads within a range of 5 bp of the annotated exon boundary (Additional file 1: Figure S1). This step captures clipped reads when part of the clipped region matches the adjacent intronic sequence, and thus the clip is not performed at the exact boundary coordinate; and it allows for additional space for potentially added sequence at the cDNA ends that could have been introduced during cloning. Further, cDNA-detector infers a consensus sequence from the clipped overhangs. It then searches the alignment for additional reads with short overhangs (1-2 bp) annotated as mismatches rather than clips, but whose overhang sequence aligns to the inferred consensus sequence at the respective boundary (Additional file 1: Figure S1). Additional evidence from reads identified in these steps is added to the clipped read count, and *P*-values are re-calculated for all candidate contaminant exons and transcripts as described above. cDNA-detector further checks whether the consensus sequence of the clipped region maps to the neighboring exon.

### cDNA source inference

Eukaryotic genomes contain endogenous retrogenes, processed (spliced) copies of genes that were reverse transcribed and re-inserted into the genomic sequence, often flanked by LINE-1 elements in the human genome [8–10]. Similar to contaminant cDNAs, these retrogenes are also intron-free and generate clipped reads at the source gene locus, and thus cDNA-detector will detect these instances. While retrogenes are rarely picked up in ChIP-seq and ATAC-seq data, they can dominate genomic sequencing strategies (whole exome and whole genome). The tool therefore attempts to distinguish vector-introduced contaminants from retrogene copies (Methods; Additional file 1: Figure S1). To infer the source of a given cDNA candidate, a database of known cloning vectors [11] as well as consensus sequences of transposable elements [12] are queried for the consensus sequences derived from clips at the 5’ and 3’ ends of candidate transcripts. In the common case where clipped sequences are too short to yield a database query result, we consider the distance of the clipped position to the annotated boundary: if the distance is ≤ 5 bp, a vector is likely the source; if the distance is > 10 bp, the cDNA candidate is deemed more likely to originate from a retrogene insertion. Cases where no clear evidence can be found (e.g. custom plasmids, short sequences) are annotated as “unknown”, and the user is encouraged to manually review potential sources. When using cDNA-detector on genomic sequence data, we recommend suppressing the “retrocopy” output, such that only potential vector cDNA candidates are reported.

### Alignment decontamination

If potential contaminants are identified, cDNA-detector provides the option to remove contaminant reads from the alignment (Fig. 1B and Additional file 1: Figures S2A-B) to reduce the risk of spurious coverage peak and variant calls in downstream analysis. In addition to obvious contaminant clipped reads as identified above, cDNA-detector further classifies all reads in candidate cDNAs as either genomic or ambiguous (Additional file 1: Figure S2B; Methods). Genomic reads span the exon boundary and properly map to the adjacent intron sequence; ambiguous reads do not overlap the boundary but instead map entirely inside the exon, and could thus originate from either true signal or the contaminant. In the decontamination step, cDNA-detector removes all *bona fide* contaminant reads. For ambiguous reads, the tool first calculates the fraction of contaminant-to-genomic reads at the exon boundaries. Next, a similar fraction of ambiguous reads inside the exon is discarded randomly. This approach ensures that any true signal obscured by cDNA contamination remains in the decontaminated sample. With this strategy, contaminants can be effectively removed from alignments, revealing true signal previously obscured by contamination (Fig. 1C; Additional file 1: Figure S2C).

## Results

### cDNA-detector performance

**Fig. 2:**
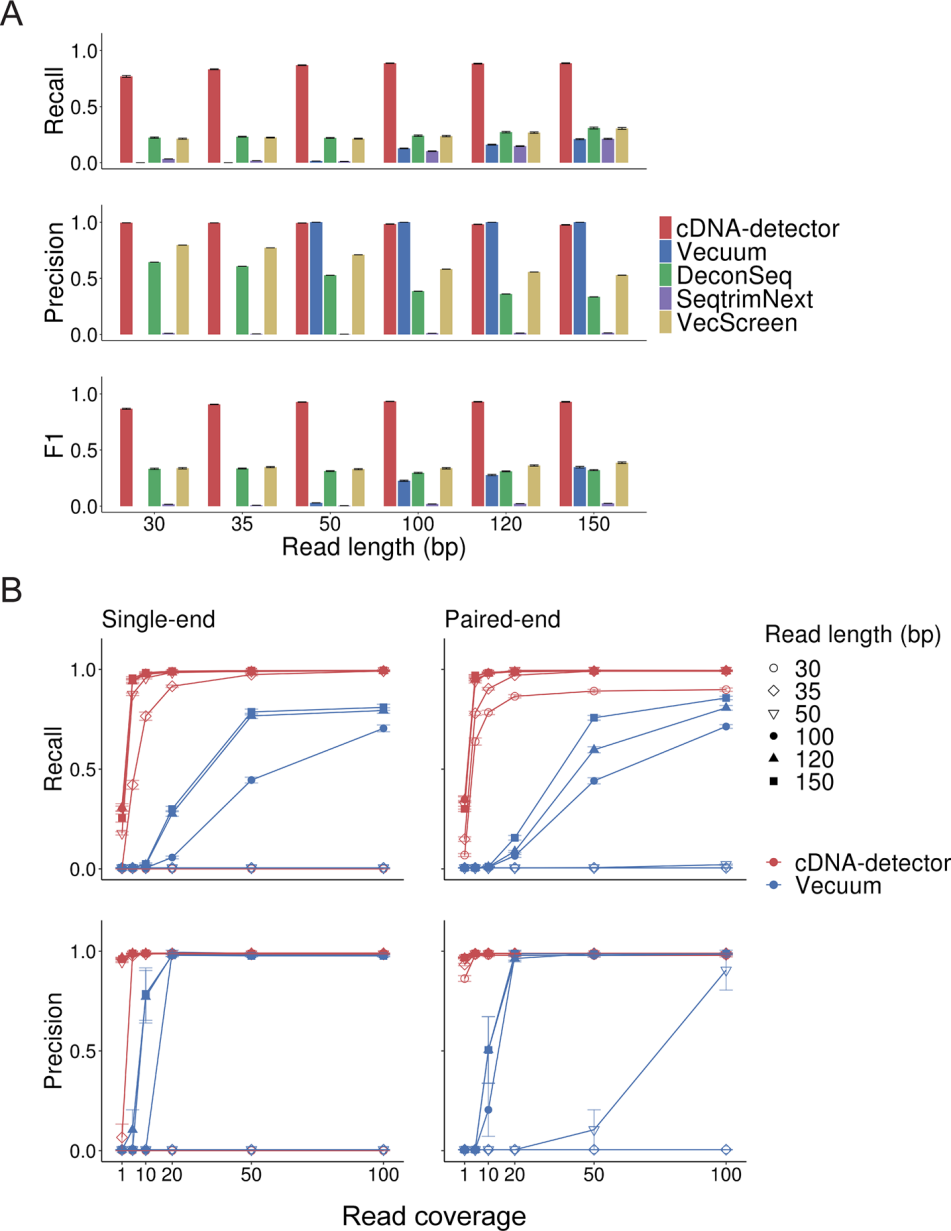
Performance of cDNA-detector. **A** Recall, precision and F1 score for cDNA-detector and related tools for detection of spiked-in contaminant reads (150 bp paired-end reads) in a simulated data set. Error bars indicate standard errors. Sample size n = 10 for each experiment. **B** Recall and precision of cDNA-detector and Vecuum on single-end (left) and paired-end (right) sequencing experiments, depending on read coverage. Error bars indicate standard errors. Sample size n = 10 for each library type and read length

Several methods exist to detect vector contamination in NGS libraries, including Vecuum [4], SeqTrimNext [13], DeconSeq [14] and VecScreen [7]. We evaluated cDNA-detector’s sensitivity (recall) and precision in light of these existing methods by generating sets of simulated cDNAs with different read lengths, distinct sequencing strategies (single vs. paired-end) and variable coverage, and introducing these into a contamination-free ATAC-seq experiment (2 × 38 bp reads, 43,344,211 reads total; Methods). Measuring the identification of mapped contaminant paired-end reads, cDNA-detector outperformed all methods in both precision and recall, with outstanding performance at short read lengths (> 70% for all read lengths; Fig. 2A). We further performed an in-depth comparison of cDNA-detector with Vecuum, the only other tool with functionality to identify and remove the actual DNA insert. We expanded our experiments to spike-in contaminants at varying coverage levels for single-end and paired-end libraries. Although both methods exhibited very high precision (> 0.95 for contaminants with $\ge$ 20 × coverage and read length $>$ 50 bp, cDNA-detector had much higher recall in both single-end and paired-end low-coverage experiments (Fig. 2B). However, cDNA-detector and Vecuum failed on very short, single-end reads because the aligner [15] with default parameters does not produce clipped reads when the read length is ≤ 30 bp.

We also applied cDNA-detector to the human whole-exome sequencing dataset from the original Vecuum publication [3, 4]. cDNA-detector detected contamination with *MTOR* cDNA in the same three of the eight exomes (Additional file 1: Figure S3A). In addition, cDNA-detector discovered *TMEM138* cDNA, which could be traced to a vector construct used in the same research group [16], supporting its validity. cDNA-detector further identified additional cDNAs of likely retrogene origin (Additional file 1: Figure S3A).

The optional second step of cDNA-detector removes inferred cDNA reads from alignment files. To test the impact of the clean-up step on downstream analysis, we compared peak calls from original, spike-in contaminant experiments (Methods) and decontaminated libraries. After discarding of contaminant reads, most spuriously gained peak calls were lost again, and cDNA-detector’s decontamination strategy was more efficient than Vecuum (Additional file 1: Figure S3B). Importantly, we also show that cDNA-detector’s decontamination step does not affect non-contaminant regions and true structural variant or short variant calls (Additional file 1: Figure S4 and Additional file 2: Table S1).

### Resource consumption

To test cDNA-detector’s compute resource consumption, we used a human exome alignment [3] with 300 M reads. From this exome, we randomly sampled different read numbers and ran cDNA-detector on a single core on a Linux high-performance cluster with 72 processors and 251 GB RAM. For small datasets (< 50 M aligned reads), the cDNA detection step was typically completed within 15 min and used ≤ 1 GB memory. cDNA detection on the full exome took about 44 min and 2 GB memory (Additional file 1: Figures S5A-B). For large datasets, including whole genome alignments, cDNA-detector can be run in multi-thread mode to decrease processing time, but requires additional memory (Additional file 1: Figures S5C-D). With its moderate resource footprint, cDNA-detector can easily be integrated into existing NGS processing pipelines as an additional quality control step.

### cDNA contamination in public databases

**Fig. 3:**
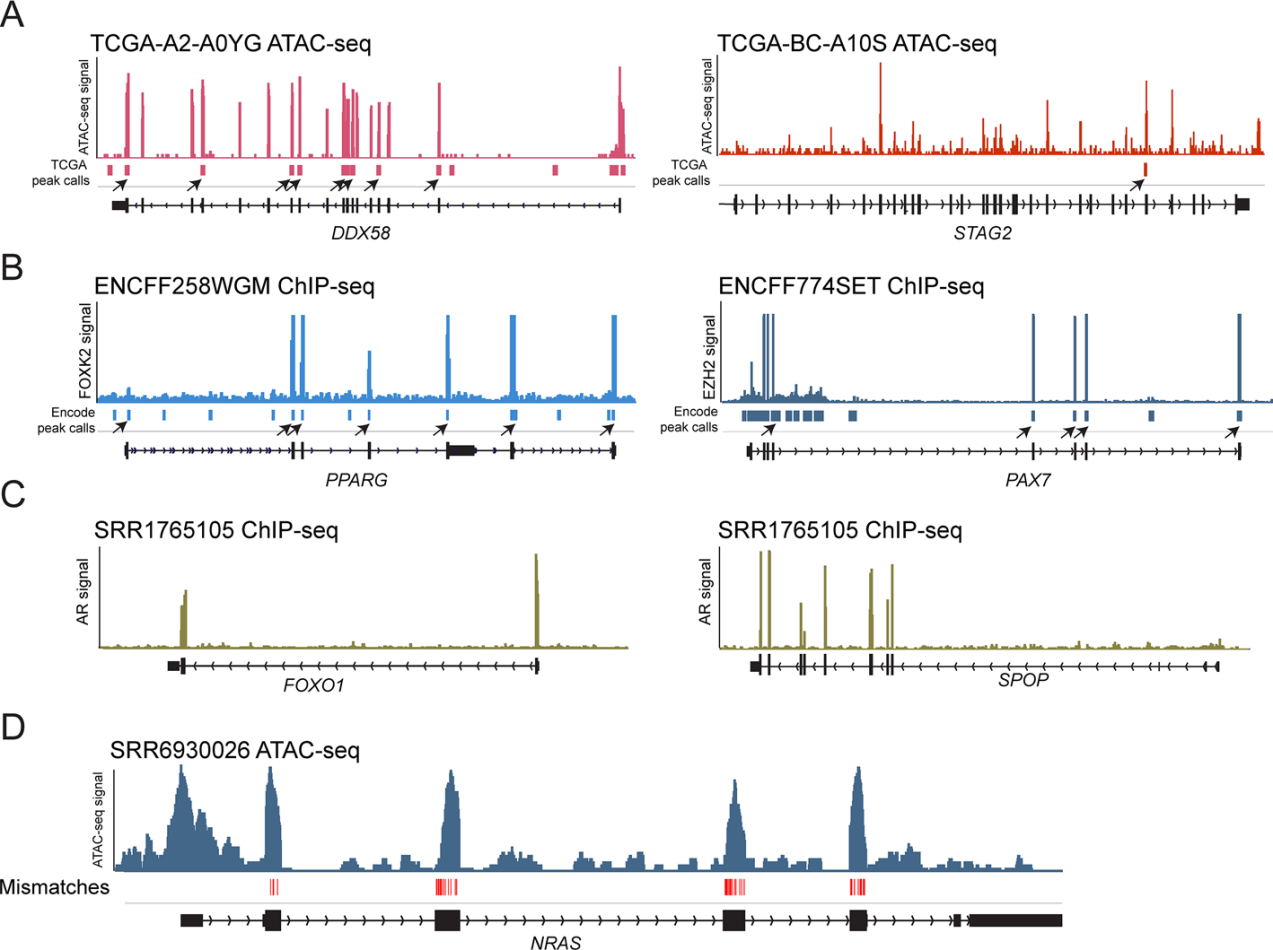
cDNA contamination in published datasets. **A** Examples of ATAC-seq signal from two primary tumor samples from the TCGA [5] showing contamination with *DDX58* (encoding the antiviral innate immune response receptor RIG-I; left) or the cohesin component *STAG2* (right). True signal would be expected at the promoter and potential intragenic regulatory elements, but not over all exons. Arrowheads indicate spurious signal peak calls caused by contaminant reads over exons (black boxes in gene track; peak calls obtained from ref [5]). **B** cDNA contamination with *PPARG* in a FOXK2 ChIP-seq experiment in HEK293T cells and *PAX7* in an EZH2 ChIP-seq experiment in HUVEC cells from the ENCODE project. Arrowheads indicate official ENCODE peak calls due to contaminant signal over exons. **C** Examples of cDNA contamination with prostate cancer genes *FOXO1* and *SPOP* in an androgen receptor (AR) ChIP-seq experiment performed in the prostate cancer cell line C2-4 [36, 37]. **D** Example of transduced human *NRAS* cDNA in a mouse ATAC-seq experiment in cell line ICC2.7

We applied cDNA-detector to several large public datasets with different types of high-throughput sequencing experiments to assess potential cDNA cross-contamination. Among 402 ATAC-seq experiments generated from primary tumors in The Cancer Genome Atlas [5], we identified 69 samples (17%) with suspected exogenous cDNA contamination. Contaminants included the cancer genes *KRAS* (2 samples, including one sample identified by manual review), *STAG2* (35 samples), *DDX58* (25 samples; *DDX58* encodes RIG-I), and *SNAP25* (9 samples), some of which can be traced to other projects from the same laboratory [17, 18] (Fig. 3A; Additional file 3: Table S2). Inspection of consensus ATAC-seq peak calls provided with the study revealed that at least some contaminant exons caused spurious peak calls that could affect downstream analyses (Fig. 3A). Sixteen cDNAs (in 41 samples) were detected in 14,485 ChIP-seq, ATAC-seq and other DNA-seq experiments from the Encyclopedia of DNA Elements (ENCODE) consortium (Fig. 3B; Additional file 4: Table S3). Some of these represent intentionally introduced genes, such as *TERT* for cell line immortalization, or *KLF4* and *MYC* in reprogrammed iPS cells (Additional file 1: Figure S6). Other genes (*PPARG, PAX7*) could not be traced to intentional transduction in the cell lines in which they were detected and are likely cross-contaminants. Peak calls generated from these experiments and available for public use are affected by these exogenous cDNAs where they contribute signal in unexpected genomic locations (Fig. 3B). We further performed cDNA detection on a random sampling of 619 human sequencing tracks from 317 projects listed in the NCBI’s Sequence Read Archive (SRA). Fifteen samples with 39 cDNAs were identified in this dataset (Fig. 3C; Additional file 5: Table S4). Detected cDNAs again included intentionally introduced transgenes, but also contaminants that could be traced to other projects in the same laboratory (e.g. refs.[19–22]; Fig. 3C). A sampling of 71 mouse genome sequencing tracks from 36 projects in SRA yielded three cDNAs. These include intentionally introduced truncated *Sun1* and *NRAS* of human origin [23], demonstrating that cDNA-detector is mismatch-tolerant and can detect instances of cross-species cDNA contamination (Fig. 3D; Additional file 6: Table S5). No candidate vector-introduced cDNAs were detected in 324 exomes from the Cancer Cell Line Encyclopedia (CCLE [24]; data not shown). Of note, only a small subset of the contaminants in these public datasets were identified by Vecuum (Methods; Additional file 7: Table S6).

## Conclusions

cDNA-detector is a highly sensitive and accurate method to identify cDNA inserts in NGS libraries and remove contaminant reads if desired. cDNA-detector does not remove vector backbone sequences unmapped to the reference genome; however, other methods exist for this purpose (e.g. DeconSeq, SeqtrimNext). Another limitation of cDNA-detector is the dependence on a pre-defined gene model to detect cDNA inserts. For newly or incompletely assembled genomes, a gene model can be approximated by mapping genes of a closely related species. Similarly, custom amplicons used in the laboratory or sequencing facility should be added to the gene model to optimize detection sensitivity.

Our results highlight that intentional and unintentional cDNA contamination is pervasive in published sequencing experiments, including some widely used, highly cited datasets, yet remains largely unnoticed. cDNA contaminants are shown to directly affect signal peak calls, and may contribute to other spurious findings. In some cases, specific genes detected as contaminant cDNAs could be traced to other published studies from the submitting laboratory, highlighting the risks of intra-lab contamination. This cross-contamination raises potential issues with keeping specific research constructs confidential and private when necessary. In addition to laboratory protocol improvements, we thus recommend careful review and computational decontamination of high-throughput genomic sequencing data for cDNA contaminants as an essential part of any sequence processing pipeline. Our two-step method consisting of detection and decontamination of cDNA allows users to review potential cDNA candidates before deciding to remove them from data, reducing the risk of accidental removal of true variants.

## Methods

### Method outline

The general framework of cDNA-detector consists of three steps: 1. Preparation of a gene model file, for example all exons for a given species and genome assembly, 2. detection of potential cDNA in a BAM-format alignment file, 3. optional clean-up of identified cDNA sequencing reads from the alignment (Fig. 1B; Additional file 1: Figures S1 and S2A).

### Generation of gene model file

An annotation file containing genomic coordinates for exon positions for a given species and assembly is required to run cDNA-detector. A processing script to generate such gene models from GTF format annotation files is part of the cDNA-detector distribution. Pre-generated models for the human (hg19 and hg38) and mouse exome (mm10 and mm39) are provided with the tool. Users may consider adding entries for specific transcript variants (such as truncated genes), custom amplicons, or non-coding RNAs that are suspected or known contaminants.

### Detection of candidate cDNA in DNA sequencing experiments

#### cDNA source reads at exon boundaries

Soft-clipped reads as identified by a read’s CIGAR string (S) in the BAM file are considered potential contaminant reads. In addition, cDNA-detector looks for reads with mismatches just past the exon boundary inside the intron (by MD tag). Reads whose overhang into the intron sequence is an exact substring of the consensus sequence of other soft-clipped reads in this location are also counted as possible cDNA reads. Consensus sequences are generated by aligning the intron overhang of all clipped reads, including a base with ≥ 80% frequency in the consensus.

#### Statistical identification of candidate cDNAs

To evaluate whether the number of soft-clipped boundary-spanning reads for a given exon exceeds the number expected by chance, we build a background expectation model by using all soft-clipped reads overlapping any exon region boundary as defined in the gene model. We then test for significant enrichment of soft-clipped reads, as calculated above, for each single exon boundary genomic position. We define the total number of reads overlapping a specific exon boundary, including genomic reads from the target experiment properly mapped to the adjacent intron, as ${n}_{t}$. The total number of soft-clipped and overhang reads is defined as ${n}_{c}$. The total number of soft-clipped reads in all exon regions is ${N}_{c}$, and the total number of all reads in all exon regions is defined as ${N}_{t}$. We use a beta-binomial model to calculate the probability of observing at least ${n}_{c}$ reads at a given exon boundary by chance (Eq. 1):

1 $$P\left({X\ge n}_{c}\right)=1-\sum\limits_{x=0}^{{n}_{c}-1}\left(\genfrac{}{}{0pt}{}{{n}_{t}}{x}\right)\frac{B(\alpha +x, {n}_{t} -x + \beta )}{B( \alpha , \beta )}$$

For $$n_c >0 $$ $$X$$ in {$${n}_{c}, {n}_{c}+1, \dots , {n}_{t}$$}, where α = $${N}_{c}$$, β = $${N}_{t}-$$ $${N}_{c}$$ + 1 and *B*(α, β) is the Beta function.

An exon with contaminant cDNA will have soft-clipped reads at both of its boundaries. We therefore combine the *P*-values for both exon boundaries for all exons using the harmonic mean *P*-value [25] (to account for dependence between the two *P*-values). Finally, all combined exon-wise *P*-values are corrected with the Benjamini–Hochberg procedure [26]. Exons with corrected *P*-value (Q-value) ≤ 0.05 are considered candidate cDNA-exons. To further increase confidence that a flagged exon originates from cDNA, the overhang consensus sequence of soft-clipped reads at both exon boundaries will be compared to the sequence of the neighboring exon(s). If at least one substring match is found, exons are kept for further analysis. Finally, transcripts with ≥ 30% of exons passing the above criteria will be reported. Because the background model is built on the total number of clipped reads at exon boundaries, it is recommended to remove adapter sequences before alignment to increase sensitivity especially for low-concentration contaminants (Additional file 1: Figure S7).

#### cDNA source inference

cDNA-detector attempts to identify the source of a candidate cDNA to distinguish endogenous (retrocopy) and exogenous (vector) origins. Source inference is performed by querying the overhang sequence of the 5’ and 3’ ends of a candidate cDNA against the NCBI UniVec database (https://www.ncbi.nlm.nih.gov/tools/vecscreen/univec/) and Repbase database of human and mouse known repeat sequences [12]. The highest-scoring match with E-value ≤ 10 is taken as probable origin. Accordingly, a “vector” is reported if the top match stems from UniVec and “retrocopy” is inferred if this hit comes from RepBase. In addition, we require a distance of > 5 bp for a repeat match from the clipped position for a “retrocopy” assignment, as these genes often carry adjacent UTR sequence [27]. In case where no suitable match is found, we use the distance from the exon boundary to the clip as estimate: if the distance is ≤ 5 bp, a cloned vector sequence is more likely (“vector-likely”); if the distance is greater than 10 bp, we infer a possible retrogene (“retrocopy-likely”); otherwise the source is labeled “unknown”. Because source inference strongly depends on the length of the overhang sequence available for BLAST query, it is thus most reliable for experiments with long read lengths.

### Removal of contaminant reads from alignment

After the candidate cDNA contamination detection step, a new alignment file can be generated with candidate contaminant reads removed. This step is straightforward for soft-clipped reads at flagged exon boundaries, as these are very likely contaminants. However, exogenous DNA will also be present as fully aligned reads inside exon regions without overlapping boundaries. In some cases, such as a first exon, these reads can overlap with actual ChIP-seq or ATAC-seq signal derived from the proximal promoter. To address this issue, cDNA-detector assigns reads in candidate cDNA exon regions into three classes: (1) clipped reads and their mates clearly obtained from cDNA contamination (${R}_{c}$); (2) reads derived from the genome aligned across an exon boundary without soft-clips or mismatches, neighboring exon homology, or a proper mate mapped outside the exon (${R}_{g}$); and (3) reads which cannot be unambiguously assigned to either class (${R}_{a}$) due to full genomic alignment inside an exon region.

For each candidate contaminant exon, cDNA-detector will first calculate the ratio of ${R}_{c}$ to all unambiguous reads (${R}_{c}+{R}_{g}$), and then remove the same fraction of ambiguous reads fully mapped inside the exon regions (${R}_{a}$):

$$R_{na} = nint\left( { \frac{{R_{c} }}{{R_{g} + R_{c} }}*R_{a} } \right)$$

where ${R}_{na}$ is the number of ambiguous reads to remove, *nint* is the nearest integer function. ${R}_{na}$ reads are then randomly selected and removed from this exon. After removal of contaminant reads in all flagged exons, a separate “clean” BAM file is written to a specified output location.

#### Performance

##### cDNA simulation experiments for sequencing strategy and read length

To evaluate detection performance, we generated simulated contaminating cDNAs by randomly selecting 100 cDNAs sequences from the CCDS database [28] for each of 10 simulation experiments. Selected cDNAs were “cloned” in silico into the pLX307 vector sequence (http://www.addgene.org/117734/), by replacing the luciferase gene with the corresponding tested cDNA. We then randomly simulated artificial paired-end or single-end reads with read lengths 30 bp, 35 bp, 50 bp, 100 bp, 120 bp, 150 bp, and fragment size 350 bp for paired-end experiments, from the vector with insert, to an average target coverage of 100 × . Simulated reads were aligned to the hg38 human genome assembly with bwa mem [15]. Aligned reads were then added to a T47D ATAC-seq experiment with *ERBB2* reads from an intentionally introduced construct removed with cDNA-detector. For experiments with < 100 × coverage, we randomly downsampled to the desired target coverage from this alignment. cDNA-detector was run with default settings to discover simulated cDNA contamination.

##### Resource consumption

In order to test the time and memory usage and multi-threaded performance of cDNA-detector, we randomly extracted reads to the desired read number from exome SRR1819826 [3]. Time and memory consumption were assessed for cDNA-detector with default settings.

#### Comparison with related tools

For comparison of cDNA-detector with Vecuum (1.0.1) [4], SeqtrimNext (2.0.68) [13], DeconSeq (0.4.3) [14] and VecScreen (BLAST 2.9.0 +) (https://www.ncbi.nlm.nih.gov/tools/vecscreen/), we used the simulation strategy outlined above. For SeqtrimNext, DeconSeq, VecScreen, original FASTQ or FASTA files were used as input. Alignments (BAM) were input into Vecuum and cDNA-detector. Due to different functionality of these tools, we measured the ability to identify mapped contaminant reads with the following metrics: Recall was defined as (called true simulated mapped reads)/(true simulated mapped reads); precision was defined as (called true simulated mapped reads)/(called mapped reads); F1 was defined as (2 × precision × recall)/(precision + recall). Performance to detect cDNA inserts (only Vecuum and cDNA-detector) was evaluated with recall defined as (called true cDNAs)/(true spiked-in cDNAs), precision as (called true cDNAs)/(called cDNAs) and F1 as (2 × precision × recall)/(precision + recall). Due to possible identical alignments for reads derived from homologous genes, a homolog was also counted as true positive if identified as cDNA candidate.

We further compared cDNA-detector to Vecuum using the exome data used in the respective publication (SRP055482; [3]). FASTQ files were aligned to the hg19 genome assembly with bwa mem with the default settings [15]. cDNA-detector was run with –num_initial_potential_cdna 5000 (allowing a larger number of initial cDNA candidates for evaluation) to detect cDNA contamination. Candidate cDNAs were manually reviewed for presence of soft-clipped reads and other evidence in the Integrative Genome Viewer (IGV; [29]).

We also applied Vecuum to the samples from the TCGA, NCBI SRA (human samples only) and ENCODE in which cDNA-detector identified candidate cDNAs. Vecuum run with default parameters (minimal match length *l* = 20 and mapping quality threshold *Q* = 30) detected few cDNAs in the comparison data. Individual hits could be recovered with Vecuum with more relaxed parameters (-l15 -Q1 for TCGA ATAC-seq; -l 10 -Q1 for NCBI SRA and ENCODE data), although at the cost of lost specificity.

#### Assessment of downstream effect of cDNA contamination and application of cDNA-detector

For assessment of peak calling after cDNA decontamination, we used the simulated cDNA strategy described above. MACS2 (2.2.7.1) [30] was applied to call peaks in the original, contaminated, and decontaminated (by either cDNA-detector or Vecuum) samples. Differential peaks were counted with BEDTools (v2.30.0) [31].

For assessment of variants of cDNA contamination, we generated simulated cDNAs with one random point mutation (limited to chromosome 10) and added them to the human HG002 whole-genome sequencing sample from the Genome In A Bottle project [32]. Then we applied cDNA-detector to identify and remove cDNAs. Bcftools (1.9) [33] was used to call variants in the original HG002, contaminated, and decontaminated samples.

#### Effect of adapter sequences on cDNA-detector

We aligned reads from the T47D ATAC-seq sample before adapter removal to the hg38 human genome, then simulated cDNAs as described above, and compared performance metrics to the same sample after adapter removal.

#### Application to public datasets

We applied cDNA-detector snapshot 26 to three large public data sets using the Terra.bio platform and on local compute: 402 ATAC-seq experiments from primary tumors from TCGA [5], 14,485 ChIP-seq, ChIA-PET, ATAC-seq and DNase-seq tracks from ENCODE [34] with read length > 30 bp, 324 exomes from the Cancer Cell Line Encyclopedia [24], and 317 human and 36 mouse studies from the NCBI’s SRA repository (see references in Additional files 5 and 6: Tables S4 and S5). Adapters were removed from TCGA ATAC-seq libraries before running cDNA-detector, and results were inspected manually. Only high-confidence cDNA candidates are reported. SRA studies were selected based on the availability of BAM-format files and restricted to ChIP-seq, ATAC-seq and DNase-seq experiments with sequence read length > 30 bp. We then randomly selected one or two (if the study contained more than one experiment) samples from those projects. SRA files were downloaded, then converted to BAM format with sam-dump (https://ncbi.github.io/sra-tools/sam-dump.html) and samtools [35] with default parameters. Analysis with cDNA-detector was performed as described above. Potential cDNAs were manually reviewed in IGV.

## Availability and requirements

Project name: cDNA-detector. Project home page: https://github.com/rheinbaylab/cDNA-detector. Operating system(s): Any. Programming language: Python. Other requirements: Python 3.7.6 or higher, BLASTN 2.9.0. License: BSD-3-Clause License. Any restrictions to use by non-academics: none.

## Supplementary Information

**Additional file 1**. Supplementary Figures S1-S7.

**Additional file 2**. Effect of contaminant cDNA reads and decontamination with cDNA-detector on structural variant calling in HG002 WGS.

**Additional file 3**. Identified cDNAs in TCGA ATAC-seq samples.

**Additional file 4**. Identified cDNAs in samples from ENCODE.

**Additional file 5**. Identified cDNAs in select human samples from the NCBI SRA.

**Additional file 6**. Identified cDNAs in select mouse samples from the NCBI SRA.

**Additional file 7**. cDNAs detected by Vecuum in select samples from TCGA, ENCODE and NCBI SRA datasets.

## Data Availability

cDNA-detector is available from GitHub at https://github.com/rheinbaylab/cDNA-detector, Docker Hub at https://hub.docker.com/r/rheinbaylab/cdna-detector and as a workflow in Terra.bio (https://portal.firecloud.org/?return=terra#methods/cDNAdetector/cDNAdetector/26; https://portal.firecloud.org/?return=terra#methods/cDNAdetector_clean/cDNAdetector_clean/2 (requires login)).

## Abbreviations

ATAC-seq: Assay for transposase-accessible chromatin using sequencing
CCDS: The Consensus Coding Sequence
CCLE: Cancer cell line encyclopedia project
ChIA-PET: Chromatin Interaction Analysis by Paired-End Tag Sequencing
ChIP-seq: Chromatin immunoprecipitation followed by sequencing
CIGAR: Compact idiosyncratic gapped alignment report
ENCODE: Encyclopedia of DNA elements
iPS: Induced pluripotent stem cell
LINE-1: Long interspersed nuclear element-1
NCBI: National Center for Biotechnology Information
NGS: Next-generation sequencing
RAM: Random-access memory
SRA: Sequence read archive
TCGA: The Cancer Genome Atlas
UTR: Untranslated region

## References

[1] Kim J, Zhao B, Huang AY, Miller MB, Lodato MA, Walsh CA (2020). APP gene copy number changes reflect exogenous contamination. Nature.

[2] Lee M-H, Siddoway B, Kaeser GE, Segota I, Rivera R, Romanow WJ (2018). Somatic APP gene recombination in Alzheimer’s disease and normal neurons. Nature.

[3] Lim JS, Kim W-I, Kang H-C, Kim SH, Park AH, Park EK (2015). Brain somatic mutations in MTOR cause focal cortical dysplasia type II leading to intractable epilepsy. Nat Med.

[4] Kim J, Maeng JH, Lim JS, Son H, Lee J, Lee JH (2016). Vecuum: identification and filtration of false somatic variants caused by recombinant vector contamination. Bioinformatics.

[5] Corces MR, Granja JM, Shams S, Louie BH, Seoane JA, Zhou W (2018). The chromatin accessibility landscape of primary human cancers. Science.

[6] Sequence Cleaner [Internet]. [cited 2021 Jul 13]. https://sourceforge.net/projects/seqclean/

[7] VecScreen: Screen for Vector Contamination. [cited 2021 Jul 13]. https://www.ncbi.nlm.nih.gov/tools/vecscreen/

[8] Esnault C, Maestre J, Heidmann T (2000). Human LINE retrotransposons generate processed pseudogenes. Nat Genet.

[9] Wei W, Gilbert N, Ooi SL, Lawler JF, Ostertag EM, Kazazian HH (2001). Human L1 retrotransposition: cis preference versus trans complementation. Mol Cell Biol.

[10] Kaessmann H, Vinckenbosch N, Long M (2009). RNA-based gene duplication: mechanistic and evolutionary insights. Nat Rev Genet.

[11] The UniVec Database [Internet]. [cited 2021 Jul 13]. https://www.ncbi.nlm.nih.gov/tools/vecscreen/univec/

[12] Bao W, Kojima KK, Kohany O (2015). Repbase Update, a database of repetitive elements in eukaryotic genomes. Mob DNA.

[13] Falgueras J, Lara AJ, Fernández-Pozo N, Cantón FR, Pérez-Trabado G, Claros MG (2010). SeqTrim: a high-throughput pipeline for pre-processing any type of sequence read. BMC Bioinform.

[14] Schmieder R, Edwards R (2011). Fast identification and removal of sequence contamination from genomic and metagenomic datasets. PLoS ONE.

[15] Li H, Durbin R (2009). Fast and accurate short read alignment with Burrows-Wheeler transform. Bioinformatics.

[16] Lee JH, Silhavy JL, Lee JE, Al-Gazali L, Thomas S, Davis EE (2012). Evolutionarily assembled cis-regulatory module at a human ciliopathy locus. Science.

[17] Mazumdar C, Shen Y, Xavy S, Zhao F, Reinisch A, Li R (2015). Leukemia-associated cohesin mutants dominantly enforce stem cell programs and impair human hematopoietic progenitor differentiation. Cell Stem Cell.

[18] Chen YG, Kim MV, Chen X, Batista PJ, Aoyama S, Wilusz JE (2017). Sensing self and foreign circular RNAs by intron identity. Mol Cell.

[19] Pan C-W, Jin X, Zhao Y, Pan Y, Yang J, Karnes RJ (2017). AKT-phosphorylated FOXO1 suppresses ERK activation and chemoresistance by disrupting IQGAP1-MAPK interaction. EMBO J.

[20] Yang Y, Blee AM, Wang D, An J, Pan Y, Yan Y (2017). Loss of FOXO1 cooperates with TMPRSS2–ERG overexpression to promote prostate tumorigenesis and cell invasion. Cancer Res.

[21] Shi Q, Zhu Y, Ma J, Chang K, Ding D, Bai Y (2019). Prostate cancer-associated SPOP mutations enhance cancer cell survival and docetaxel resistance by upregulating Caprin1-dependent stress granule assembly. Mol Cancer.

[22] Huang SN, Williams JS, Arana ME, Kunkel TA, Pommier Y (2017). Topoisomerase I-mediated cleavage at unrepaired ribonucleotides generates DNA double-strand breaks. EMBO J EMBO.

[23] Seehawer M, Heinzmann F, D’Artista L, Harbig J, Roux P-F, Hoenicke L (2018). Necroptosis microenvironment directs lineage commitment in liver cancer. Nature.

[24] Ghandi M, Huang FW, Jané-Valbuena J, Kryukov GV, Lo CC, McDonald ER (2019). Next-generation characterization of the cancer cell line encyclopedia. Nature.

[25] Wilson DJ. The harmonic mean p-value for combining dependent tests. 10.1101/171751 10.1073/pnas.1814092116 PMC6347718 30610179

[26] Benjamini Y, Hochberg Y (1995). Controlling the false discovery rate: a practical and powerful approach to multiple testing. J R Stat Soc.

[27] Casola C, Betrán E (2017). The genomic impact of gene retrocopies: what have we learned from comparative genomics, population genomics, and transcriptomic analyses?. Genome Biol Evol.

[28] Pujar S, O’Leary NA, Farrell CM, Loveland JE, Mudge JM, Wallin C (2018). Consensus coding sequence (CCDS) database: a standardized set of human and mouse protein-coding regions supported by expert curation. Nucl Acids Res.

[29] Robinson JT, Thorvaldsdóttir H, Winckler W, Guttman M, Lander ES, Getz G (2011). Integrative genomics viewer. Nat Biotechnol.

[30] Zhang Y, Liu T, Meyer CA, Eeckhoute J, Johnson DS, Bernstein BE (2008). Model-based analysis of ChIP-Seq (MACS). Genome Biol.

[31] Quinlan AR, Hall IM (2010). BEDTools: a flexible suite of utilities for comparing genomic features. Bioinformatics.

[32] Zook JM, McDaniel J, Olson ND, Wagner J, Parikh H, Heaton H (2019). An open resource for accurately benchmarking small variant and reference calls. Nat Biotechnol.

[33] Li H (2011). A statistical framework for SNP calling, mutation discovery, association mapping and population genetical parameter estimation from sequencing data. Bioinformatics.

[34] Davis CA, Hitz BC, Sloan CA, Chan ET, Davidson JM, Gabdank I (2018). The encyclopedia of DNA elements (ENCODE): data portal update. Nucl Acids Res.

[35] Li H, Handsaker B, Wysoker A, Fennell T, Ruan J, Homer N (2009). The sequence alignment/map format and SAMtools. Bioinformatics.

[36] Zhao J, Zhao Y, Wang L, Zhang J, Karnes RJ, Kohli M (2016). Alterations of androgen receptor-regulated enhancer RNAs (eRNAs) contribute to enzalutamide resistance in castration-resistant prostate cancer. Oncotarget.

[37] Zhao Y, Wang L, Ren S, Wang L, Blackburn PR, McNulty MS (2016). Activation of P-TEFb by androgen receptor-regulated enhancer RNAs in castration-resistant prostate cancer. Cell Rep.

