# Supplementary material for: cDNA-detector: detection and removal of cDNA contamination in DNA sequencing libraries
Source: BMC Bioinformatics. 2021 Dec 24;22:611. doi: 10.1186/s12859-021-04529-2 (PMC8709999; doi:10.1186/s12859-021-04529-2)
Supplement: Supplementary file 1 — Additional file 1. Supplementary Figures S1-S7. [file 12859_2021_4529_MOESM1_ESM.pdf]

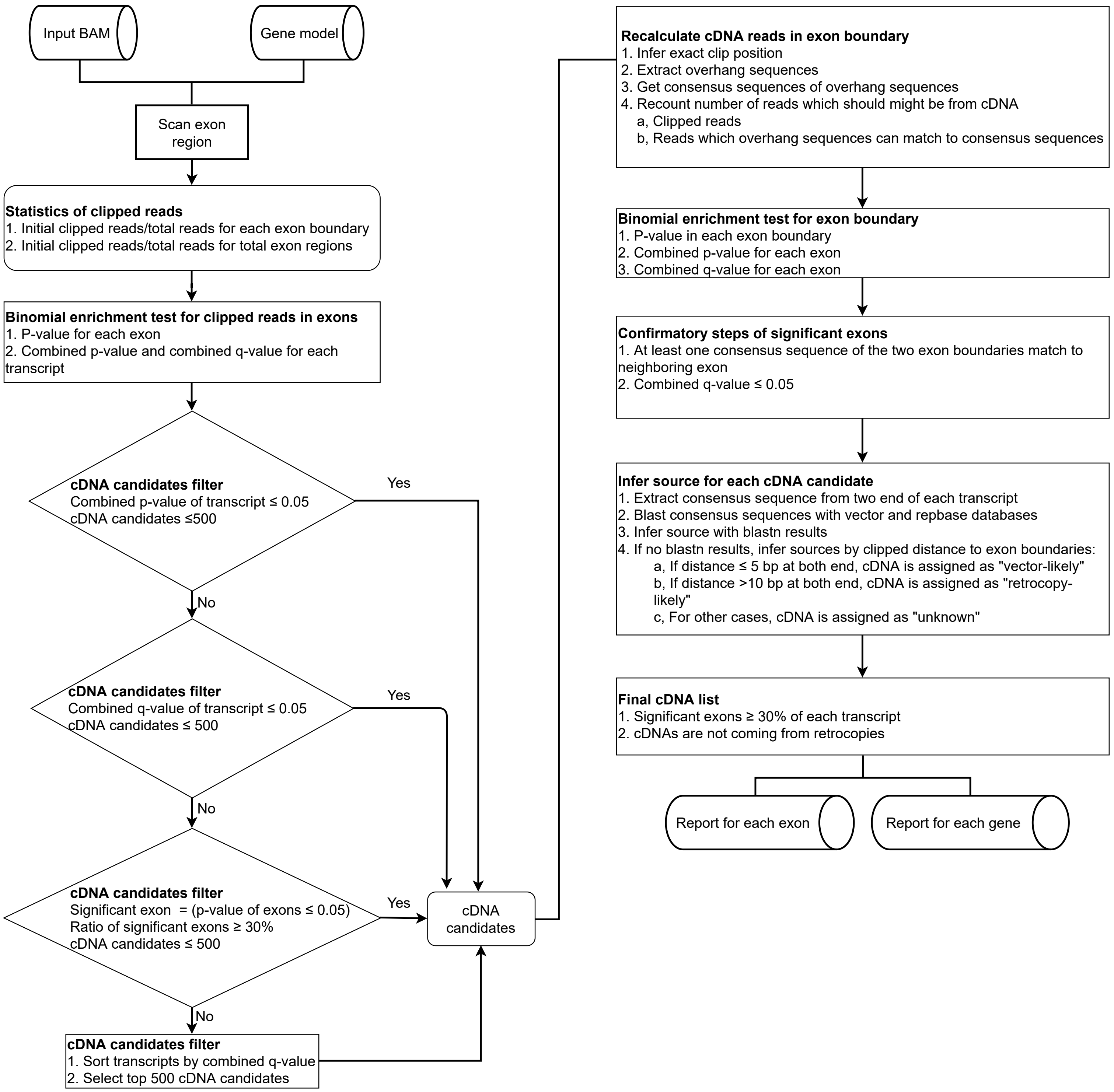

Figure S1: Flowchart detailing the cDNA-detector detection step.

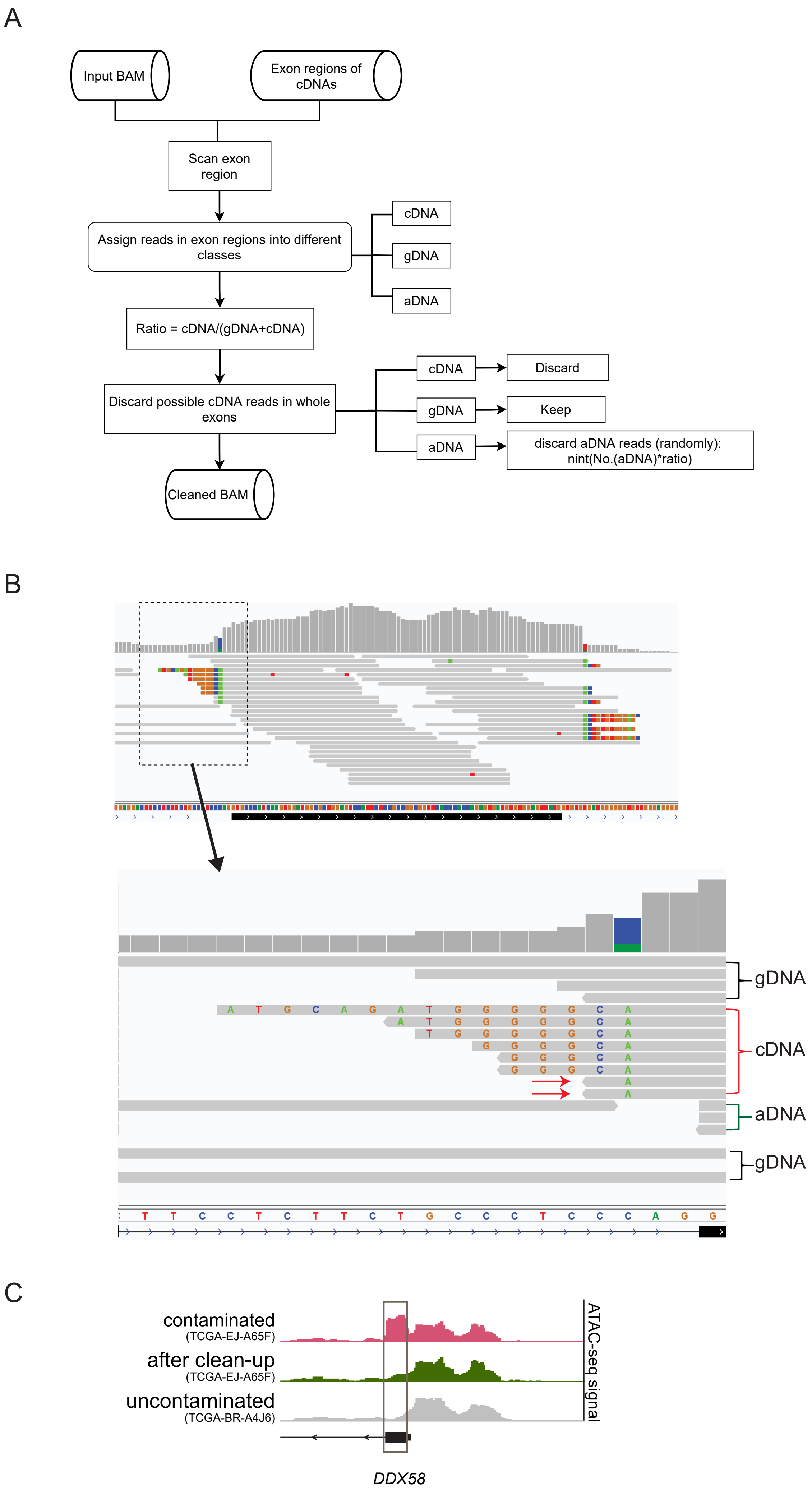
A
Input BAM
Exon regions of cDNAs
Scan exon region
Assign reads in exon regions into different classes
cDNA
gDNA
aDNA
Ratio = cDNA/(gDNA+cDNA)
Discard possible cDNA reads in whole exons
cDNA
Discard
gDNA
Keep
aDNA
discard aDNA reads (randomly): nint(No.(aDNA)*ratio)
Cleaned BAM
B
gDNA
cDNA
aDNA
gDNA
C
contaminated
(TCGA-EJ-A65F)
after clean-up
(TCGA-EJ-A65F)
uncontaminated
(TCGA-BR-A4J6)
ATAC-seq signal
DDX58

Figure S2: Decontamination of cDNA reads from NGS libraries. A, Flowchart depicting the decontamination step. **B**, Three classes of reads at an exon boundary are considered in the decontamination step: cDNA, genomic DNA (gDNA), and ambiguous reads originating from either cDNA or genomic signal (aDNA). Red arrows show examples of cDNA reads with mismatches instead of clips. **C**, Example of an ATAC-seq experiment contaminated with *DDX58* cDNA and cDNA-detector “decontamination”. Contaminant signal is clearly visible over the first exon, overlapping real ATAC-seq signal from the transcription start site (top, pink track). After the decontamination step, the enrichment over the exon is removed while preserving the ATAC-seq peak (green), comparable to an uncontaminated sample (grey). Gene transcribed from the reverse strand.

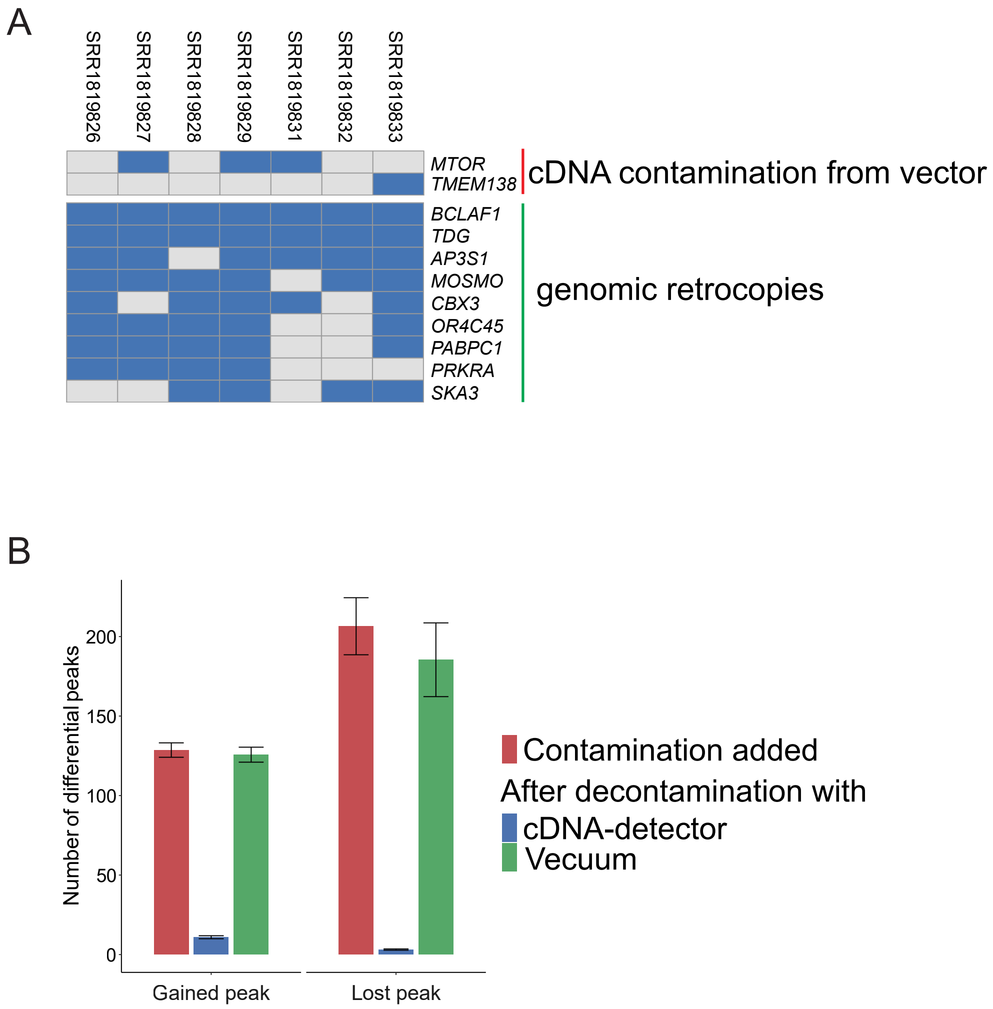

Figure S3: Comparison between cDNA-detector and Vecuum. A, cDNA identified by cDNA-detector in a human whole-exome sequencing dataset. Identification of known vector contamination (*MTOR*), previously unknown vector contamination (*TMEM138*) and genomic retrocopies of processed genes by cDNA-detector in the dataset presented in the Vecuum publication (Kim et al, *Bioinformatics,* 2016). Blue represents detection of cDNAs, grey indicates no hit. **B**, Downstream effect of cDNA contamination and after decontamination by cDNA-detector and Vecuum on peak calling. After simulated cDNAs (150 bp paired-end reads) were inserted into a cDNA-free ATAC-seq sample, cDNA-detector and Vecuum were applied to remove contaminants. Peaks were called by MACS2 in the original, contaminated, decontaminated samples, and differential peaks were counted by comparing with peak counts from the original (uncontaminated) sample. Error bars indicate standard errors. Sample size equals 10 for each condition.

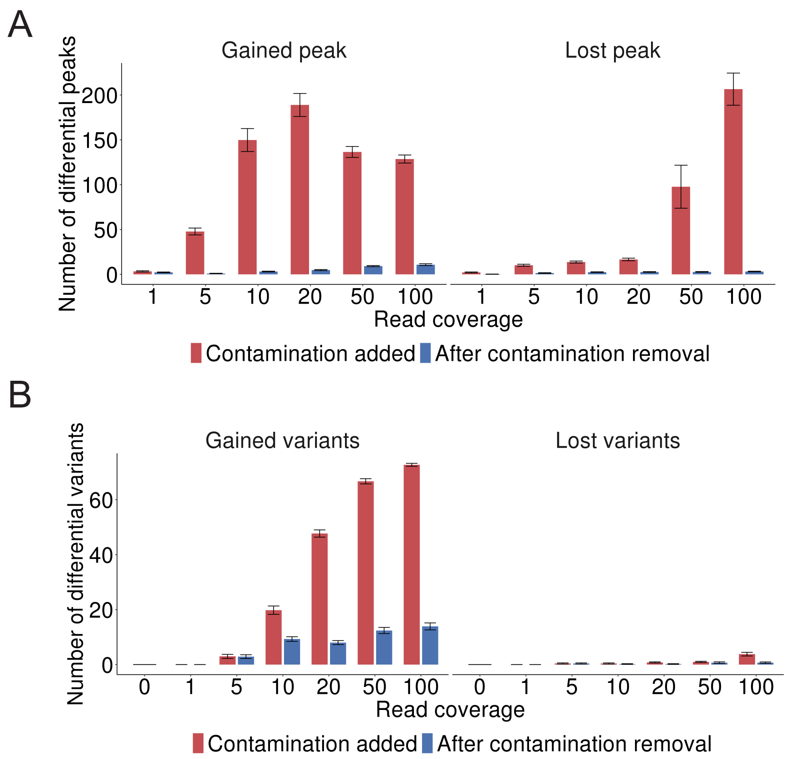

Figure S4: Assessment of downstream effect of cDNA contamination and application of cDNA-detector. A, Effect of cDNA contamination and decontamination by cDNA-detector on ATAC-seq peaks, depending on different coverage of inserted simulated cDNAs (100 simulated cDNAs per experiment, paired-end reads with read length 150 bp). Gained and lost peaks are compared to the original cDNA-free T47D ATAC-seq experiment. Error bars indicate standard errors. Sample size equals 10 for each condition. **B**, Impact of cDNA contamination and decontamination with cDNA-detector on SNP calling depending on different coverage of inserted simulated cDNAs (see above). Gained and lost variants are compared to those called in the original human HG002 whole-genome sequencing library. Error bars indicate standard errors. Sample size equals 10 for each condition.

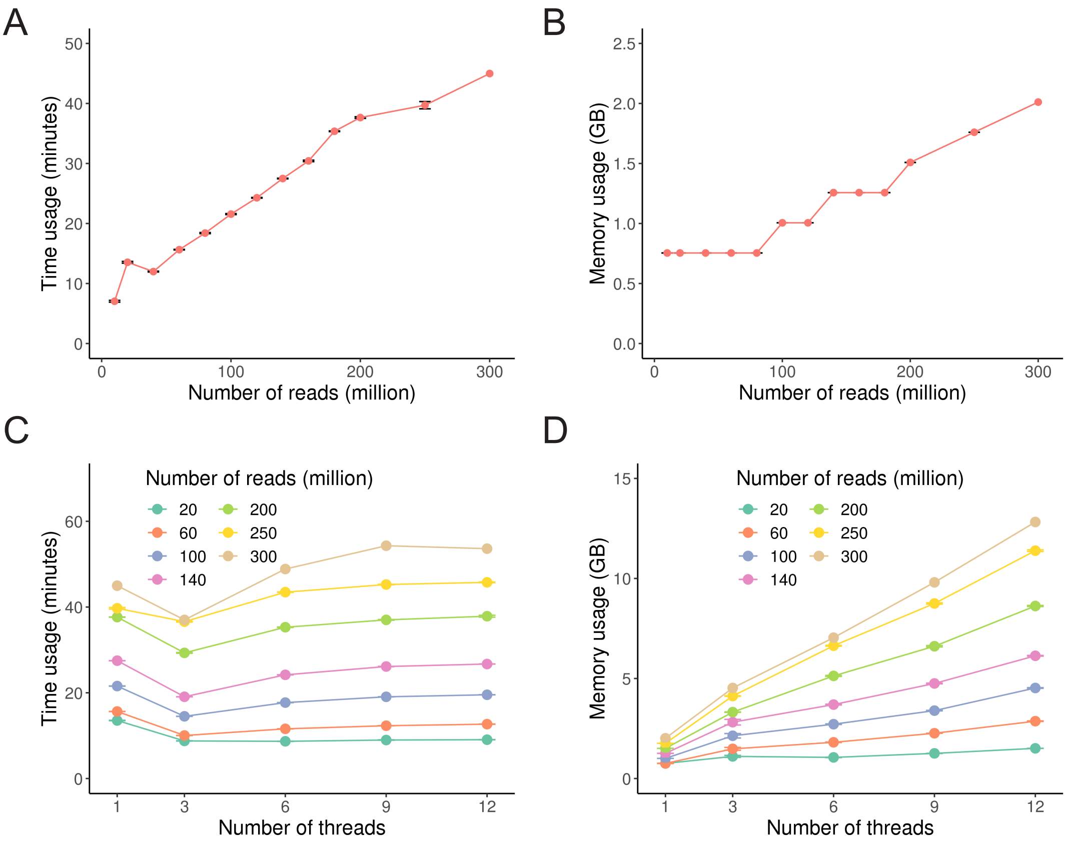

Figure S5: Resource consumption of cDNA-detector. A, Runtime of the cDNA-detector detection step depending on the number of reads in the alignment. **B,** Memory usage in GB of the detection step for different number of reads. **C-D**: Time (**C**) and memory (**D**) usage for different read numbers when using multiple threads. The error bars indicate standard errors. Sample size n = 10 for each simulation (except the full data set of 300 M reads).

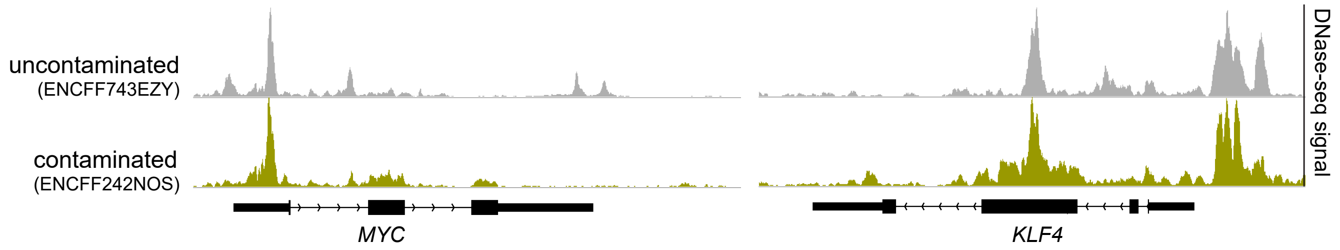

Figure S6: cDNA identified in a reprogrammed iPS cell line. *MYC* and *KLF4* cDNA in L1-S8R cells (yellow) reprogrammed with *OCT4, SOX2, KLF4, cMYC,* and uncontaminated control cell line PC-3 (grey).

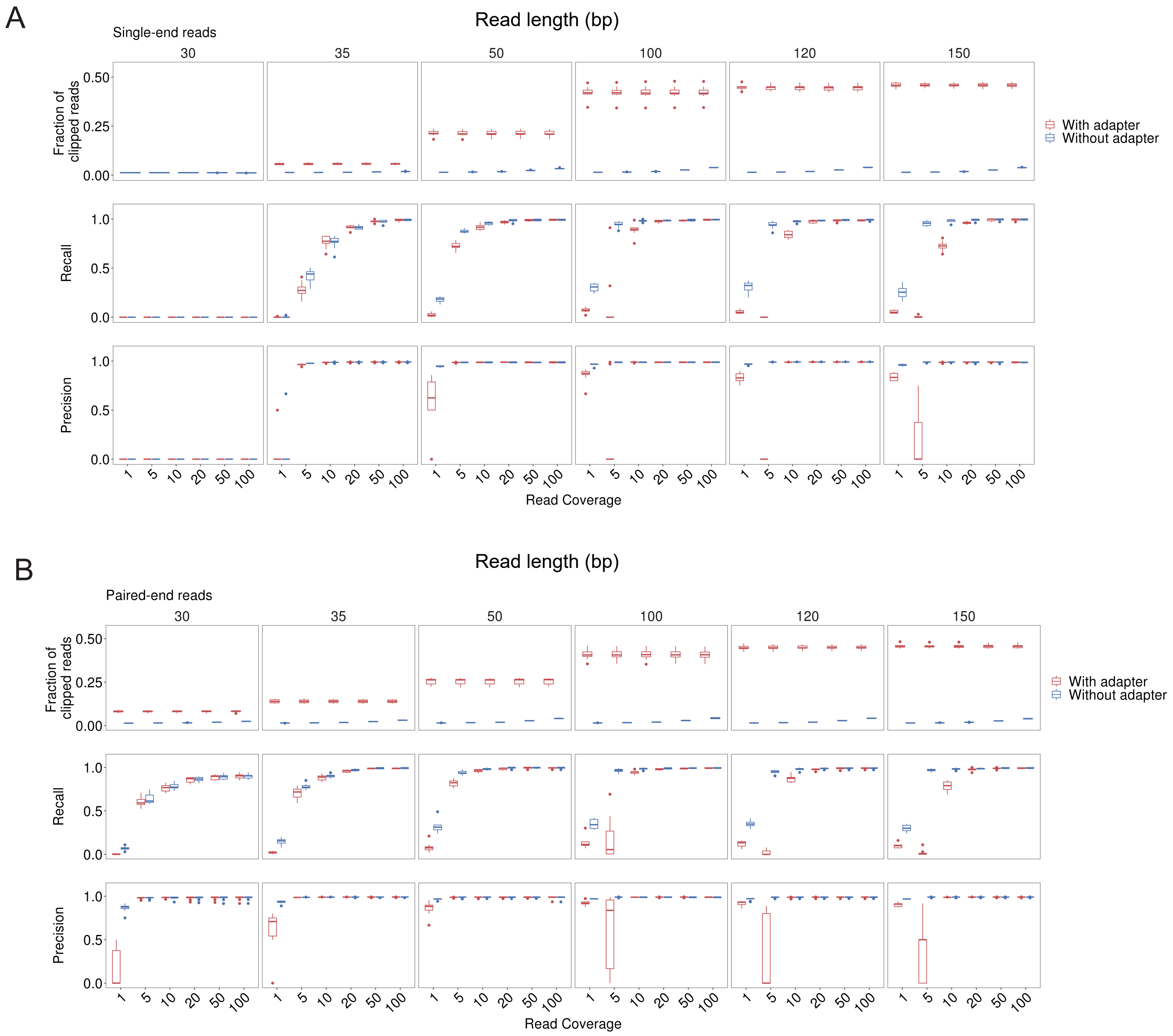

Figure S7: Effect of adapter sequences on cDNA-detector performance. Performance assessment (recall and precision) in spike-in experiments in a T47D ATAC-Seq sample with and without adapter sequences. Experiments were performed as described, different read lengths, coverage and library strategies (**A**, single-end; **B**, paired-end). "Fraction of clipped reads" indicates the fraction in exon regions. Sample size equals 10 for each condition.
